# Factors predicting the active treatment of renal angiomyolipoma: 30 years of experience in two tertiary referral centers

**DOI:** 10.3389/fsurg.2023.1094806

**Published:** 2023-05-11

**Authors:** Arnaud Daché, Richard Fatica, Brian R. Herts, Gordon McLennan, Erick M. Remer, Georges-Pascal Haber, Idir Ouzaid

**Affiliations:** ^1^ Department of Urology, Bichat Claude Bernard Hospital, University Paris-Cité, Paris, France; ^2^ Cleveland Clinic, Glickman Urological and Kidney Institute, Cleveland, OH, United States

**Keywords:** angiomyolipomas, nephron-sparing surgery, angioembolization, active surveillance, renal tumors

## Abstract

**Introduction and objective:**

This study aimed to identify clinical features representing predictive factors of active treatment (AT) compared to active surveillance (AS) for renal angiomyolipoma (AML).

**Patients and methods:**

From 1990 to 2020, patients referred to two institutions for a renal mass and diagnosed with an AML based on typical features on CT were included in the analysis. The study population was divided into two groups based on the treatment received: active surveillance (AS) or active treatment (AT). Age, gender, tuberous sclerosis syndrome, tumor size, contralateral kidney disease, renal function, year of diagnosis, and symptoms at presentation were assessed as potential predictive factors of active treatment using a logistic regression model in univariate and multivariate analyses.

**Results:**

In total, 253 patients (mean age 52.3 ± 15.7 years; 70% women; 70.9% incidentally diagnosed) were included in the analysis. One hundred and nine (43%) received AS, whereas 144 (57%) were actively treated. For univariate analysis, age, tuberous sclerosis complex syndrome, tumor size, symptoms at presentation, and contralateral kidney disease were found to be predictors of AT. Only tumor size (*p* < 0.001) and the year of diagnosis (*p* < 0.001) remained significant for multivariable analyses. The likelihood of being managed with AS evolved over the study period and was 50% and 75% when diagnosed before and after 2010, respectively. With respect to size, 4-cm and 6-cm tumors had a probability of 50% and 75% of being treated with AS, respectively.

**Conclusion:**

The present analysis from a high-volume institution provides evidence that the management of renal masses with typical radiological features of AML has markedly changed over the last three decades with a trend toward AS over AT. Tumor size and the year of diagnosis were significant factors for the treatment strategies.

## Introduction

Angiomyolipoma (AML) is the only benign renal tumor that can be confidently diagnosed by cross-sectional imaging, with fat density within a lesion as a diagnostic hallmark (1, 2). Regions with Hounsfield unit densities of −80 or less are diagnostic of gross fat within a lesion and considered diagnostic of AML. Diagnosis is more difficult for the less common variant of AML with minimal fat at imaging. For these lesions, findings of more than 20 pixels with attenuation less than −20 HU or more than 5 pixels with attenuation less than −30 HU, when present, were shown to have a positive predictive value of 100% but are not sensitive for a diagnosis of minimal-fat AML (3).

As a nonmalignant tumor, its prognosis is related to potential bulking effect symptoms including flank pain, hematuria, and hemorrhage. Spontaneous renal hemorrhage, also known as Wunderlich's syndrome, is a rare but potentially life-threatening complication that occurs in up to 10% of patients, especially in the setting of larger tumors (4–6).

As a result, surgical excision was considered the main treatment option in young patients with large and/or symptomatic diseases (7). Recently, minimally invasive treatment options have been described including cryoablation and angioembolization (2, 8, 9). The obvious benefits of active surveillance (AS) include maximum preservation of normal functional renal parenchyma and avoidance of the morbidity associated with active treatment (AT).

Most practice guidelines for AML are empirically derived upon outcomes of retrospective studies, with a limited sample size over a short period. Moreover, given disease incidence and prognosis, randomized trials are not likely to be conducted. Consequently, evidence is lacking on whether these tumors should be treated pre-emptively or treatment be deferred until the patient becomes symptomatic.

High-volume referrals to our institutions have allowed for the acquisition of a large data set on AML. This study aimed to review our institutional 30 years’ experience in the management of these tumors and identify clinical drivers for AT as opposed to AS.

## Patients and methods

In this HIPAA-compliant retrospective study, we reviewed the charts of patients referred to our institution from 1990 to 2020 for renal mass, with a radiological diagnosis of AML based on CT findings (identification of fat-containing mass). Patients were grouped, according to their treatment, into either AS or AT. AS consisted of 6-month, 12-month, and then yearly monitoring, which consisted of a physical exam and one of the following imaging modalities: ultrasound, computed tomography (CT), or magnetic resonance imaging (MRI). AT included open, laparoscopic, and robotic partial or radical nephrectomy, angioembolization, and cryoablation. The diagnosis of AML was confirmed at final pathology only for patients who underwent surgery or percutaneous biopsy.

Then, clinical features predicting the likelihood of one treatment option over the other were assessed. Age, gender, tuberous sclerosis complex syndrome, tumor size, year of diagnosis, contralateral kidney disease, chronic kidney disease (CKD) stage, and clinical presentation were assessed as potential factors associated with a higher likelihood of AT.

### Statistical analyses

Data are presented as means ± standard deviations (SDs) or proportions when appropriate. Categorical and continuous variables were assessed using *χ*^2^ and Student’s *t* tests, as appropriate. Univariable and multivariable cox regression models were used to determine the association between active treatment and the clinical features of AML. Logistic fit and inverse prediction of AS by significant factors on the multivariable analysis were plotted. All *p*-values were two-sided, and statistical significance was defined as a *p* < 0.05. Statistical analyses were performed using SPSS (IBM SPSS Statistics for Windows, Version 24.0, IBM Corp., Armonk, NY, USA).

## Results

In total, 253 patients met the inclusion criteria and were included in the analysis. The mean age of the study population was 52.3 ± 15.7 years. Seventy-seven percent of patients were women. At presentation, 70.9% were asymptomatic. Hematuria (14.1%) and flank pain (9.7%) were the most commonly reported symptoms. Table 1 summarizes the baseline patient characteristics.

**Table 1:** Patients’ characteristics.

	*N* = 253	
Age, years, mean ± SD	52.3 ± 15.7	
**Gender, *n* (%)**		
Male	56	(22.1)
Female	197	(77.9)
**Side, *n* (%)**		
Right	111	(43.9)
Left	117	(46.3)
Bilateral	25	(9.8)
**Tuberous sclerosis syndrome, *n* (%)**		
No	225	(88.9)
Yes	28	(11.1)
Tumor size, cm ± SD	5.5 ± 5.1	
**Presentation, *n* (%)**		
Incidental	179	(70.9)
Flank pain	24	(9.7)
Hematuria	36	(14.1)
Retroperitoneal hemorrhage	5	(1.8)
Other	9	(3.5)

SD, standard deviation.

**Figure 1:**
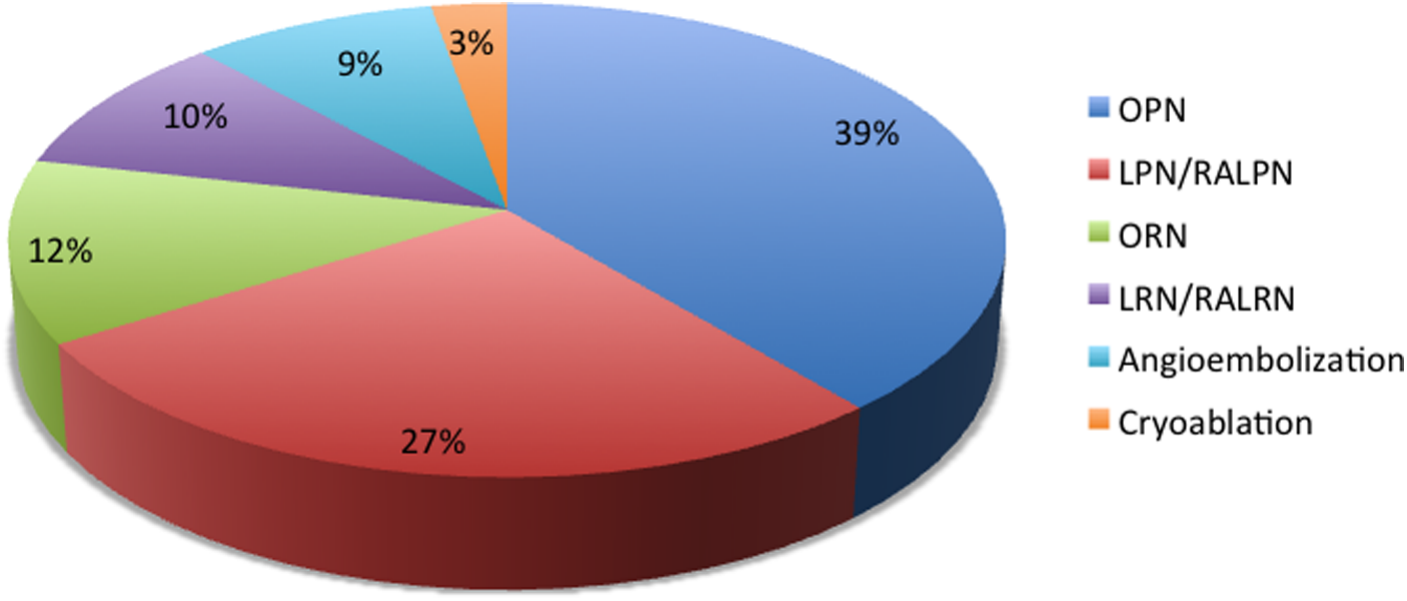
Active treatment options. OPN, open partial nephrectomy; LPN, laparoscopic partial nephrectomy; RALPN, robot-assisted laparoscopic partial nephrectomy ORN, open radical nephrectomy; LRN, laparoscopic radical nephrectomy; RALRN, robot-assisted laparoscopic radical nephrectomy.

Of the selected patients, 109 (43%) and 144 (57%) underwent AS and AT, respectively. Surgical excision was the most frequent treatment option (88%), followed by angioembolization (9%) and cryoablation (3%). Figure 1 details the different treatment options.

On univariate analyses, age at diagnosis, TSC, tumor size, symptoms at presentation, contralateral kidney disease, and year of diagnosis were all associated with AT (Table 2). All of these factors, except tumor size and year of diagnosis, were not significant in multivariate analysis (Table 3).

**Table 2:** Univariable analysis of the likelihood of active treatment vs. surveillance.

	Surveillance (*n* = 109)		Active (*n* = 144)		*p*-Value
Age, years, mean ± SD	56.7 ± 14.6		48.7 ± 15.3		0.0001
**Gender, *n* (%)**					
Female	85	(77.9)	111	(77.1)	0.865
Male	24	(22.1)	33	(22.9)	
**Tuberous sclerosis syndrome, *n* (%)**					
No	103	(94.5)	122	(84.7)	0.006
Yes	6	(5.5)	22	(15.3)	
Tumor size, cm ± SD	2.57 ± 2.3		7.85 ± 5.6		0.0001
**Presentation, *n* (%)**					
Incidental	85	(77.9)	94	(65.6)	0.0375
Symptomatic	24	(22.1)	50	(34.4)	
**Contralateral kidney disease**					
Yes	19	(17.4)	44	(30.6)	0.0154
No	90	(82.6)	100	(69.4)	
**Chronic kidney disease stage**					
1	38	(34.8)	48	(33.3)	0.831
2	52	(47.8)	79	(54.6)	
3	18	(16.3)	17	(12.1)	
4	1	(1.1)	0	(0)	

SD, standard deviation.

**Table 3:** Multivariable analysis of factors predicting active treatment.

Multivariate analysis	*p*-value
Age	0.564
Tuberous sclerosis syndrome	0.723
Tumor size	<0.001
Symptoms at presentation	0.121
Contralateral kidney disease	0.284
Year of diagnosis	<0.001

**Figure 2:**
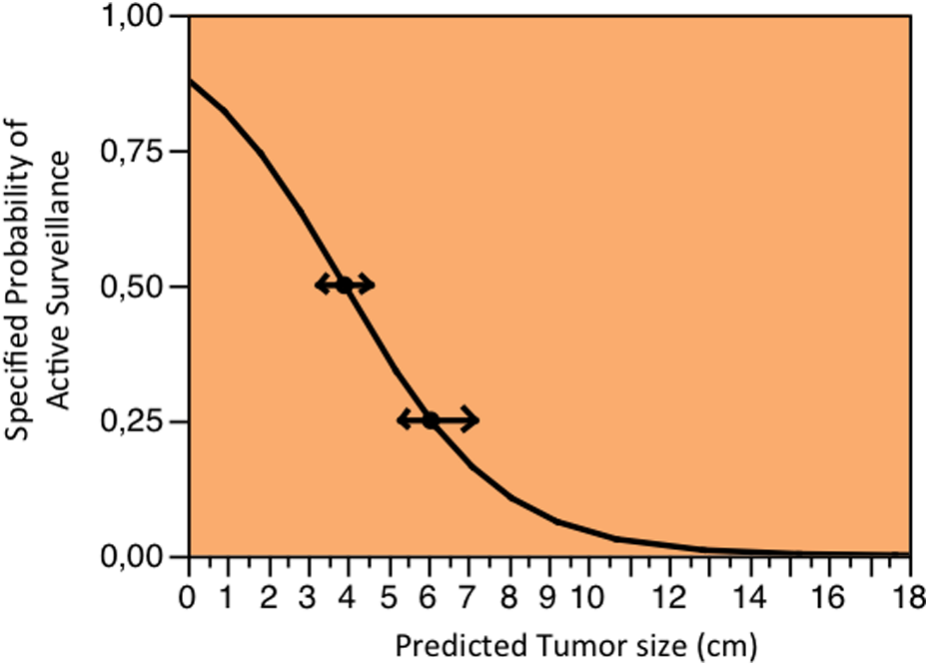
Specific probability of being treated with active surveillance according to the tumor size. A patient diagnosed with 4-cm and 6-cm tumors had a probability of 50% and 25% (arrows) of being treated with active surveillance, respectively.

Based on the logistic fit plot analyses, recent tumors were more likely to be treated with AS. For example, the probability of being managed with AS was 50% and 75% before and after 2010, respectively. The probability of being treated with AS was also inversely proportional to tumor size. A patient diagnosed with 4-cm and 6-cm tumors had a probability of 50% and 25% of being treated with AS, respectively (Figure 2).

## Discussion

According to current guidelines (Level C recommendation), primary indications for intervention for patients with an AML include symptoms such as pain, bleeding, or suspected malignancy (7). Thus, prophylactic intervention seems justifiable for large tumors (the recommended threshold of intervention is >4 cm), women of childbearing age, or patients in whom follow-up or access to emergency care may be inadequate (7, 10).

With respect to these guidelines, our results suggest that only tumor size was associated with AT on multivariate analysis. In addition, the year of diagnosis was also highly predictive of AT. Conversely, gender, age, and symptoms were not associated with AS.

Mues and colleagues investigated factors predicting surgical intervention or embolization in a series of 91 AMLs (11). They used a regression model to assess predictors of AT in patients treated initially with AS. However, only three patients failed AS in the analysis, which might have undermined the power of their statistical model. We found that size coded as a continuous variable is strongly associated with AT, as outlined by Rosales and colleagues, who assessed factors predictive of AT in a cohort of patients with cortical renal tumors and treated with AS (12).

The radiologic appearance of the tumor is an important feature in the decision-making process and inpatient counseling. In fact, typical fatty masses are more likely to be watched, whereas fat-poor AMLs are resected because of suspicion of renal cell carcinoma (RCC). Selecting only typical AMLs on CT offsets this important confounding factor.

Like all renal masses, the trend in the management of AMLs is moving toward nephron-sparing strategies (13). Our study observed an increase in the adoption of AS since 2008. This increase can be intuitively attributed to the increased incidence of small renal masses due to the widespread use of cross-sectional imaging (14). Also, data from observational studies on RCC highlighted the potential benefits of nephron-sparing strategies on long-term cardiovascular morbidity and renal function (15). This practice can be extrapolated to all renal masses.

Our study carries some limitations. First, while it is notable that our analysis was conducted over a 30-year period, technical advances have occurred over this time that have impacted the management of AMLs. These advances have occurred not only in the fields of minimally invasive urologic surgery and minimally invasive radiological procedures but also in medical imaging. It can be estimated that CT diagnosis yields improved by 10% over the last decade (16–19). Consequently, AMLs were more likely to be treated with AT during the first period of this study, regardless of clinical presentation and size. Second, despite the high diagnostic accuracy of current cross-sectional imaging, the diagnosis of AMLs could not be confirmed in most of our AS patients since they have not systematically undergone a percutaneous renal biopsy. Also, we are cautious that some masses treated with AS without prior percutaneous biopsy might be malignant in nature since fat-containing RCC has already been reported (20). Finally, due to the retrospective design of this study, the superiority of one treatment vs. the other cannot adequately be compared because AT precedes the AS period, ultimately introducing bias. Ideally, a prospective randomized study would compare both treatment options with respect to subsequent cost and morbidity. Such a study is difficult to design due to the low incidence of AMLs and the high proportion of incidentally diagnosed AMLs. Nevertheless, it is noteworthy to assess whether the current threshold (4 cm) for AT should be revised and a larger-size threshold should be established.

## Conclusion

Our high-volume, single-institution analysis provides evidence that managing renal masses with typical radiological features of AML has changed markedly over the last three decades toward AS over AT. Tumor size and year of diagnosis were the identified significant factors in the choice of intervention or surveillance at diagnosis. Additional research areas need to address whether prophylactic intervention in patients with large asymptomatic AML is cost-effective.

## Data Availability

The raw data supporting the conclusions of this article will be made available by the authors without undue reservation.
